# Knowledge and Acceptance Towards Mammography as Breast Cancer Screening Tool Among Yogyakarta Women and Health Care Providers (Mammography Screening in Indonesia)

**DOI:** 10.1007/s13187-019-01659-3

**Published:** 2019-11-27

**Authors:** Lina Choridah, Ajeng Viska Icanervilia, Marloes Josephia Maria de Wit, Antoinette D.I. van Asselt, Wahyu Tri Kurniawan, Yusnia Irchami Fahmi, Anggraeni Ayu Rengganis

**Affiliations:** 1 Department of Radiology, Faculty of Medicine, Public Health and Nursing, Universitas Gadjah Mada, Jalan Farmako, Sekip Utara, Yogyakarta, 55281 Indonesia; 2 Department of Health Sciences, University Medical Center Groningen, University of Groningen, Hanzeplein 1, 9713 GZ Groningen, the Netherlands; 3 Philips Electronics Singapore Pte. Ltd, Lorong 1, Toa Payoh, 319763 Singapore; 4 Department of Epidemiology, University Medical Center Groningen, University of Groningen, Hanzeplein 1, 9713 GZ Groningen, the Netherlands

**Keywords:** Breast cancer, Screening, Knowledge, Acceptance, Mammography

## Abstract

Annual mammography remains the gold standard of asymptomatic breast cancer screening for women starting at the age of 40. However, Indonesia has not designated mammography as its national screening program. To help policymakers decide whether mammography should be introduced into a national program, it is important to comprehensively understand the knowledge and acceptance of both consumers and providers. A total of 25 subjects including a range of women and health care professionals (HCPs) in Yogyakarta Province were recruited using purposive, maximum variation sampling and then interviewed in-depth. The interviews were recorded and all data were taken and transcribed from the audio recording, which were subsequently translated to English and analyzed thematically. Almost all of Yogyakarta women had heard about the term of mammography. However, only few of them have let themselves be screened, mainly because of their perceived lack of urgency to screen for asymptomatic breast cancer. Another important reason was the high cost of mammography. Meanwhile, several HCPs believed that breast cancer has not been a priority for the government and hence the government limited mammography screening’s access and excluded it from the national insurance coverage. Most women in Yogyakarta have a good understanding about breast cancer screening, but their acceptance of mammography as a breast cancer screening tool is significantly influenced by high cost, limited access, and lack of urgency.

## Introduction

Globally, breast cancer is the most common cancer that occurs in women [1]. In Indonesia, 40 out of 100,000 women are newly diagnosed with breast cancer each year [2]. Furthermore, most of these women are diagnosed at a late stage which may explain why Indonesia has a higher mortality rate (16.6/100,000) compared with the global rate (12.9/100,000) [2, 3]. A generally apprehensive attitude towards cancer treatment in Indonesia, including alternative medicine seeking behavior, has also led to unfavorable clinical outcomes for cancer patients [4, 5].

Although no longer recommended by the WHO [6], breast self-examination (BSE) is still perceived as breast cancer screening and widely promoted by the Indonesian government up to now. Besides, clinical breast examination (CBE) has also currently been added in the Indonesia national guideline as a recommended breast cancer screening program [7]. On the other hand, mammography has not been designated as an organized program in this country and is not covered by the national health insurance (BPJS). This is in contrast with the American Cancer Society’s position [8] that mammography is proven to reduce mortality and remains the gold standard of breast cancer screening for women aged 40 or older [9, 10]. Furthermore, there is still a lack of clear guidelines regarding the use of mammography screening in Indonesia [11].

To assist policymakers in deciding whether mammography should be introduced to a national program, it is important to gain a comprehensive understanding of the perspectives of both consumers and providers, as well as an understanding of their acceptance towards mammography use and its perceived issues. This study aims to examine the knowledge and acceptance of community members and health care professionals (HCP) towards mammography as a screening tool.

## Methods

### Study Design and Sample

Four investigators conducted in-depth interviews in May–June 2017, with a range of women and health care providers in Yogyakarta Province, Indonesia. This province was selected because of its socio-demographic diversity and unique cultural background, which can be considered representative of Indonesia. In-depth interviews were used because this is a trusted tool in qualitative studies for the exploration of subjects’ thoughts and experiences through one-on-one interaction between the researcher and participant [12]. Ethical approval was received prior to the study from the Ethics Committees Faculty of Medicine, Universitas Gadjah Mada, Yogyakarta, Indonesia.

### Participants

A total of 25 subjects were recruited using purposive, maximum variation sampling. Purposive sampling was chosen to provide the rich information from key informants which cannot be gained from others [12]. Thus, all informants were people known by the authors for being insightful and outspoken about their thoughts. Key informants had two different backgrounds: women from the general community and health care professionals (HCPs). In the general community, women were selected to represent varying levels of experience in dealing with breast cancer (i.e., they were patient, a patient’s relative, volunteer, or had no experience), as this might influence the level of knowledge they had on mammography screening, as well as their acceptance. HCPs were chosen according to their breast cancer–related expertise in different levels of care (i.e., general practitioner, specialist doctors, nurses) to speak about their perspectives, as well as their patients’ perspective.

### Data Collection

Informed consent was obtained from all participants prior to the study. Data were collected using individual, in-depth interviews in the local language based on interview guidelines that include a comprehensive list of topics and questions related to the following themes: (1) knowledge and perception towards breast cancer and breast cancer screening, (2) acceptance towards breast cancer screening methods, (3) knowledge and acceptance on mammography, and (4) current health care system on breast cancer screening. Paraphrasing and additional questions were used to seek clarification during the interviews to ensure the rigor of research and that participants’ views were accurately reported. Prompts were only given when the interviewer deemed that it was necessary to bring the conversation back to topic or to address a certain issue. Recruitment continued until data saturation was reached.

### Analysis

All data were taken and transcribed from audio recording, which were then translated to English. Data was analyzed thematically by categorizing, summarizing, and reconstructing data while searching for patterns through the use of thematic coding [13]. The coding framework was developed in the coding-team meetings, involving all investigators (with the exception of A.D.I.A). Development of consensus coding continued until saturation was achieved (i.e., no new coding emerged from examining additional transcripts). Transcripts were coded manually by research assistants and reviewed by the principal investigator.

## Results

A total of 25 interviews were conducted with 12 general community women (3 breast cancer patients, 3 breast cancer patient relatives, 1 breast cancer organization volunteer, and 5 women without breast cancer condition or relatives) and 13 health care professionals (3 general practitioners, 2 radiologists, 2 radiation oncologists, 2 oncologists, 2 oncology nurses, 1 surgeon, and 1 hospital director). The interview results are summarized thematically in Table.1. The themes described in more detail below Table 1.

**Table 1:** Subjects’ knowledge and acceptance towards breast cancer and screening methods

1. Knowledge about breast cancer and the screening methods	
a. Breast cancer is a high burden disease	Number of cases is rising
	High mortality rate
	High cost
	Time consuming
b. Breast cancer has not been a government priority	Metabolic diseases received more attention
	Only BSE routinely promoted
	Mammography screening is not covered by BPJS
c. Various methods of breast cancer screening	BSE is perceived as the most popular screening method
	None of the participants was familiar with CBE
	Most participants had heard about mammography
2. Acceptance towards mammography screening	
a. Lack of urgency in taking mammography while asymptomatic	Not a priority because they feel healthy
	Doctor or national recommendation is required
b. Experience of mammography	Need husband’s permission
	Painful but tolerable
	Worried if the machine could transmit breast cancer
c. Mammography is too expensive	Other screening method is more affordable
	Will take mammography if it is free
	Expect mammography to be covered by BPJS
d. Limited access to mammography	Only few health care facilities with mammography
	Mammography facilities are located in the central city
	Great effort to access mammography

### Breast Cancer is a High-Burden Disease

“Breast cancer here (in Yogyakarta) is associated with death, as it is often only detected at a later stage and incurable” (Administrative officer)

All participants believed that the number of people diagnosed with breast cancer was increasing but none of them was certain about the exact number. Almost all participants would associate breast cancer with a high mortality rate and suggested various reasons that may cause this such as rapid progression of disease, late diagnosis, and lack of adequate treatment.

Most participants agreed that breast cancer is a high burden disease. They highlighted that breast cancer is a high-cost and time-consuming disease. One volunteer of a local breast cancer organization said that most patients had to pay a vast sum of money for chemotherapy drugs not covered by BPJS. In addition, the current referral system also made patients suffer as they have to go through a long administrative procedure to acquire adequate diagnosis and treatment.

### Breast Cancer Has Not Been a Government Priority

“In 2017, the MOH established a new division of “non-communicable diseases.” However, the MOH programs mainly focus on diabetes mellitus and hypertension” (General practitioner)

According to two HCPs, one obstacle in implementing mammography as a screening tool in Indonesia was the lack of government programs. They said breast cancer is not on the list of the top priorities by the government. This was illustrated by programs created by the Ministry of Health (MOH) which were mainly related to infectious diseases. A disease like cancer used to be assigned under the division of communicable diseases. It was only recently (early 2017) that the MOH established a separate division for managing non-communicable diseases, including cancer, although the new division mainly focuses on metabolic diseases such as diabetes mellitus and hypertension. Several HCPs mentioned the only type of breast cancer screening that had been promoted routinely by the government was breast self-examination (BSE).

### Various Methods of Breast Cancer Screening

“I found out about mammography from a hospital advertisement around ten years ago and I was immediately interested to try because I have family members diagnosed with breast cancer” (Housewife)

Almost all general community women were familiar with BSE locally known as sadari. Most of them knew sadari from health promotion programs conducted by primary health care or the local breast cancer organization. There was only one woman who had never heard of BSE. Several women performed BSE regularly, although they were neither assured about the correct technique nor the time to do this examination.

None of the general community women was familiar with CBE, but most of them had heard of mammography from hospital advertisements or their relatives’ experience. Only one participant had never heard of mammography. Most participants thought that mammography is the best method for detecting breast cancer. Some of them mentioned ultrasonography was also a tool for breast cancer screening.

### Lack of Urgency in Taking Mammography While Asymptomatic

“I don’t want to… I am totally fine and don’t show any symptoms, so I don’t want to do it (mammography)” (Housemaid)

Among the general community women, only one woman had undergone mammography on her own initiative, while the others said that they would it if their doctor instructed them to do so. They thought that mammography is required only if they show symptoms such as a breast lump. Furthermore, there was no government recommendation for every woman to undertake mammography, so they did not see this examination as a priority. They preferred allocating their time and resources for other, more urgent and important matters.

Several HCPs mentioned that a history of breast cancer in a family member or friend frequently became a trigger for someone to have mammography. However, among the participants who were breast cancer patients’ relatives, only one person had done mammography screening. Even so, she only did it once and did not plan to do it regularly. The other two patients’ relatives were not certain if they should conduct such a screening test.

### Experience with Mammography

“I did mammography twice… One with the old machine and one with the new machine. I felt less pain and more comfortable with the new machine” (Hospital manager)

Five out of twelve general community women had an experience in doing mammography but only three of them did it for screening purposes, whereas the others for diagnosis. They said that there was no particular cultural or religious barrier in doing mammography, although they would feel more comfortable if the machine operator was a female. One woman said that she would need her husband’s permission for having the examination, particularly if the operator was male.

Many participants, including HCPs, said that healthy patients on occasion worried or even believed that the mammography machine could actually infect them with breast cancer. Thus, if a woman was diagnosed with breast cancer after a mammography examination, she might think that she contracted it from the machine.

Due to the compression procedure of mammography, several participants stated this examination was less comfortable compared with ultrasonography. The examination was said to be rather painful but tolerable. One participant had had mammography twice: first with an analog machine and second with a digital machine. She said that the pain was reduced with the digital machine.

### Mammography is Too Expensive

“As an ordinary employee, I think mammography is very expensive. I still need to pay my daughter’s school tuition. Her education is more important. Mammography is not urgent” (Hospital officer)

Cost seems to be the main obstacle that was stopping the participants from having a mammography examination. One participant illustrated that the cost was one third of her monthly salary (500,000 IDR ≈ 35 USD) and she said that she would have mammography if it was free or covered by the insurance. Two participants revealed that they had mammography only because there was a free examination program from a hospital.

Most women would prefer BSE or CBEover mammography, because these first two methods are significantly cheaper. All HCPs believed that breast cancer screening should be covered by BPJS to encourage people to take the screening. However, one HCP thought this was not an urgent matter as she thought that mammography screening was not a critical need for the community.

### Limited Access to Mammography

“In the Yogyakarta province, there are only seven mammography machines. Most of them are in private hospitals or clinics” (Oncology surgeon)

HCP participants said that mammography could only be accessed in certain hospitals in Yogyakarta, mostly located in the central city. Furthermore, most mammography facilities were located in private hospitals or clinics. Thus, one HCP said, a person must make a great effort to access mammography including time, money, a family companion, and transportation. Therefore, he said, accessibility was another barrier for conducting mammography in Indonesia, because these facilities were not distributed evenly.

## Discussion

Despite the fact that BSE is no longer recommended as breast cancer screening by the WHO, this study revealed that it is the most familiar and commonly used breast cancer screening method by women in Yogyakarta. This finding is consistent with a previous study, which found that BSE is a preferred method as it is easy, simple, cheap, and non-invasive [14]. Another study [15] revealed that despite its popularity, most women did not know the right time and technique of doing BSE, which is also in line with our study.

In our study, almost all participants had heard about mammography and realized that mammography is a better screening tool compared with BSE and CBE. This finding was in contrast with another study in Indonesia which found only 13 from 251 women who were aware of mammography. [11] However, similar to our finding, this study also found that only a small proportion of those aware of mammography, had undergone this examination. This result is supported by another study which was performed among female health care workers. The study uncovered that 84% of female health care workers had heard of mammography, but only 9% had undergone this examination in the last year [16].

The only woman in our sample that had not heard of mammography was also the only participant with a low-level education background and living in a rural area. This may be a representative finding for this particular socio-demographic background. Her lack of knowledge about mammography strongly influenced her hesitancy to do this examination. This finding was in line with another study, which found that patients’ educational level was associated with their perception of early diagnosis and disease prevention [17].

Through in-depth interviews, we found that a history of breast cancer in a family member or friend was the main trigger for the female participants to have mammography screening. A study in Sweden found similar results in which women who underwent mammography screening were more likely to have family members or friends with breast cancer [18]. However, in our study, among three participants with such characteristics, only one took mammography screening. Although this result might not reflect the real proportion in the community, this study found that there was a lack of knowledge among patients’ family members and relatives that they are at increased risk of breast cancer and for women with significant family history of breast cancer are recommended to undergo routine screening. The American Cancer Society stated that mammography alone may not be enough as a screening tool for women with significant family history, and magnetic resonance imaging (MRI) is needed as an adjunction [8, 19].

This study found several inhibiting factors for the mammography screening including high cost, limited access, and lack of urgency. A qualitative study conducted in Iran also found several inhibiting factors which are similar to our findings in addition to the public perception of the test being inessential and expensive and the lack of mammography centers [20]. Meanwhile, another study in Sweden revealed a different main inhibiting factor: the examination itself raised concerns about breast cancer [18].

Several participants in our study highlighted the need for a government campaign to raise awareness in the community about the significance of this examination. This finding is supported by another study which revealed that it is crucial to acknowledge mammography as a government priority to increase community awareness and participation [21]. In addition, other studies in Sweden and USA found that advice from health professionals is a leading determinant of attendance for mammography screening, which is consistent with the result of this study [18].

This study revealed that cost is perceived as the main factor influencing participants’ acceptance and participation. This finding is consistent with a study in Jordan which found that cost was considered a barrier by 53.4% of the subjects [22]. However, several studies found contradicting results, where screening cost and health insurance coverage were not perceived as barriers in Saudi Arabia and Qatar because breast cancer screening is free or covered by the health insurance [23, 24]; whereas a study in Sweden discovered that even when screening was offered free of charge, the attendance rate remains the same [18].

Accessibility is another crucial factor influencing participants’ acceptance of mammography. There are only seven mammography centers which mainly are found in the Yogyakarta city center. This limited number and location make it difficult for women from rural areas to access the facilities. This finding corresponds to a study in India, which revealed that high cancer mortality is correlated to incidence rate in the rural population due to a mix of late presentation, poor socioeconomic status, and restricted access to cancer-related services [25]. In addition, a study in UK also found that mobility and accessibility negatively influence mammography acceptance among elderly women [26].

The methods used in this study allowed each participant to interpret their personal understanding of and experience on breast cancer, its screening methods, and mammography. Although this study has involved HCPs from different levels of care, which may represent the perspective of patients with varying levels of education and socioeconomic status, it is limited in terms of sociodemographic variation of the general community women as most of them come from moderate to high education levels. Thus, the sample included in our study might not fully represent the broader population. This likely occurred since the recruitment of women in the community involved those who were easily contacted and would be outspoken about their thoughts. Consequently, those women mostly came from moderate to high education levels. Therefore, further quantitative research is needed to verify the findings in this study and uncover the important themes. Future research has to not only identify barriers in the implementation of appropriate screening methods, but also how to manage and solve these barriers.

## Conclusion

This study revealed diverse perspectives toward breast cancer screening from both women and health care professionals in Yogyakarta. Most of the women already have a good understanding of breast cancer screening, including mammography, and are aware of its importance. Even so, several factors such as high cost, limited access, and lack of urgency strongly influence participants’ willingness to use mammography as a breast cancer screening method. Based on the subjects’ perspectives, to increase community’s acceptance, policymakers in Indonesia should consider including mammography in the national screening program and covering it in the national health insurance plans.
